# Supplementary material for: A Plant-Derived Antifungal Agent, Poacic Acid, Inhibits Germination and Tube Growth of Lily Pollen
Source: Plants (Basel). 2025 Oct 7;14(19):3093. doi: 10.3390/plants14193093 (PMC12526336; doi:10.3390/plants14193093)
Supplement: Supplementary file 1 [file plants-14-03093-s001.zip › Figure S1.pdf]

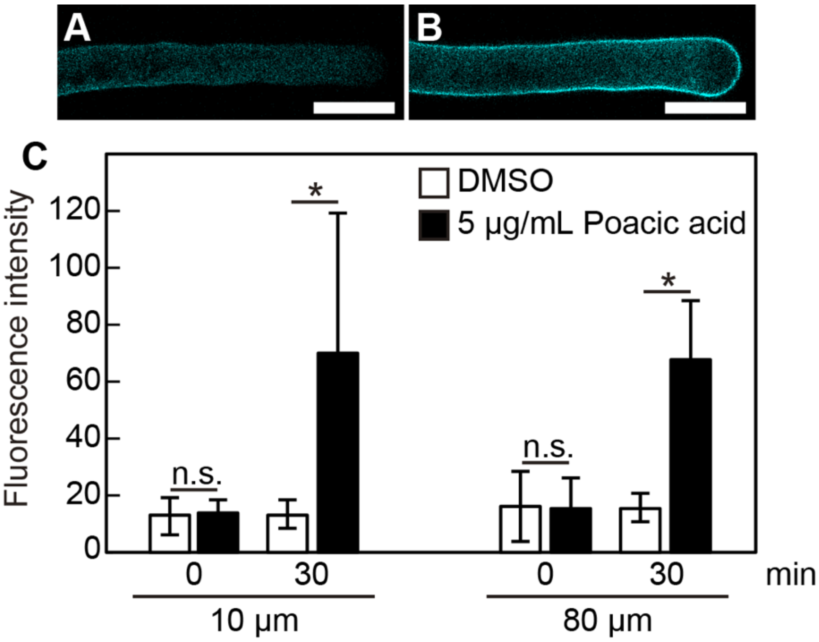

Figure S1. Enhanced callose synthesis in poacic acid-treated pollen tubes. Growing pollen tubes were transferred to media containing DMSO or 5 μg/mL poacic acid and incubated at 22°C. Pollen tubes were fixed, stained with aniline blue, and analyzed using confocal laser microscopy. (A,B) Confocal images of pollen tubes incubated for 30 min in media containing (A) DMSO or (B) 5 μg/mL poacic acid. Scale bars = 30 μm. (C) Intensities of aniline blue fluorescence at 10 μm and 80 μm from the pollen tube apex. The fluorescence intensities of at least 10 pollen tubes were measured. Statistical differences were calculated using the Student's t-test. The asterisk indicates $p < 0.01$. n.s., not significant.
